# Endothelial MicroRNA-483-3p Is Hypertension-Protective

**DOI:** 10.1155/2022/3698219

**Published:** 2022-02-17

**Authors:** Fenqing Shang, Xuan Guo, Yueer Chen, Chen Wang, Jie Gao, Ergang Wen, Baochang Lai, Liang Bai

**Affiliations:** ^1^Translational Medicine Center, Xi'an Chest Hospital, Xi'an Jiaotong University Health Science Center, Xi'an, Shaanxi 710061, China; ^2^Institute of Cardiovascular Science, Translational Medicine Institute, Xi'an Jiaotong University, Xi'an, Shaanxi 710061, China; ^3^Department of Cardiology, Xi'an No. 1 Hospital, Xi'an, Shaanxi, China; ^4^Department of Cardiology, Second Affiliated Hospital of Xi'an Jiaotong University, Shaanxi 710061, China; ^5^Department of Laboratory Animal Science, School of Basic Medical Sciences, Xi'an Jiaotong University, Xi'an, Shaanxi 710061, China

## Abstract

Hypertension is a high-risk factor for developing coronary heart disease and stroke. Endothelial dysfunction and arterial remodeling can lead to increased vascular wall thickness and arterial stiffness. Previous studies showed that microRNA-483 (miR-483) enhances endothelial cell (EC) function. Here, we investigated the protective role of miR-483 in hypertension. Data collected from two patient cohorts showed that the serum miR-483-3p level was associated with the progression of hypertension and positively correlated with vascular function. In cultured ECs, miR-483 targets a number of endothelial dysfunction-related genes, such as transforming growth factor-*β* (TGF-*β*), connective tissue growth factor (CTGF), angiotensin-converting enzyme 1 (ACE1), and endothelin-1 (ET-1). Overexpression of miR-483-3p in ECs inhibited Ang II-induced endothelial dysfunction, revealed by the decreased expression of TGF-*β*, CTGF, ACE1, and ET-1. Furthermore, miR-483-3p secreted from ECs was taken up by smooth muscle cells (SMCs) via the exosome pathway, which also decreased these genes in SMCs. Additionally, telmisartan could increase the aortic and serum levels of miR-483-3p in hypertension patients and spontaneous hypertension rats (SHR). These findings suggest that miR-483-3p exerts a protective effect on EC function during the onset of hypertension and thus may be considered a potential therapeutic target for hypertension-related cardiovascular diseases.

## 1. Introduction

Hypertension, a major contributor for developing cardiac and peripheral vascular diseases, is considered an important worldwide problem of public health [1, 2]. The key features of hypertension include endothelial dysfunction and arterial remodeling, which lead to increased vascular wall thickness and arterial stiffness [3–5]. The nitrogen oxide (NO)/endothelin-1 (ET-1) coefficient is a criterion for the risk of developing hypertension [6]. As the pathophysiological causes of hypertension are multifaceted, endothelial dysfunction can contribute to the imbalance between NO and ET-1 [4]. In addition, smooth muscle cells (SMCs) have been shown to regulate vascular homeostasis and maintain a balance between vasoconstriction and vasodilatation [7]. The cellular cross talk between endothelial cells (ECs) and SMCs is crucial for blood vessel remodeling under hypertension [8]. However, the cellular and molecular basis involved in their vasculopathic effects is little known.

MicroRNAs (miRs) are small endogenous noncoding RNAs that posttranscriptionally repress gene expression by targeting the 3′-untranslated region (3′-UTR) of mRNA transcripts [9]. Abnormal expression of miRNA is closely associated with the onset and progression of various pathological processes, including cardiovascular diseases [10–12]. New miRNA therapeutics have gained attention based on mimicking or inhibiting miRNAs. Extracellular vesicles (EVs), mainly including exosomes and microvesicles, serve as intercellular messengers and are an important mechanism in vascular health and disease [13, 14]. As an essential means of cell–cell communication, exosomes are loaded with miRNAs that are released to extracellular space [15]. For example, therapeutic delivery of miR-320d/423-5p inhibitors via engineered EV has been shown to improve vascular stiffness and alleviate the phenotype in the spontaneous hypertension rats (SHR) [16]. Therefore, research on key miRNAs has significance in therapeutic strategy of hypertension-related cardiovascular and cerebrovascular diseases.

MicroRNA-483 (miR-483), containing miR-483-3p and miR-483-5p, is encoded by a conserved sequence of insulin-like growth factor 2 (IGF2) gene [17]. The sequences of miR-483 are highly conserved among various mammalian species, such as human, rat, and mouse [10]. miR-483 is involved in the pathophysiological process of the Kawasaki disease [18], pulmonary arterial hypertension (PAH) [12], and atherosclerosis [10]. Mechanistically, miR-483 targets genes involved in transforming growth factor-*β* (TGF-*β*) signaling pathway, including transforming growth factor-*β* (TGF-*β*), TGF-*β* receptor 2 (TGF-*β*R2), Smad4, connective tissue growth factor (CTGF), and endothelin-1 (ET-1) [12, 18–21]. By suppressing these genes, miR-483 can inhibit the proinflammatory and fibrogenic responses [12, 18, 19]. miR-483 delivered to rats alleviates monocrotaline-induced pulmonary hypertension in mice [12]. Furthermore, miR-483-5p can increase LDLR expression in liver through targeting 3′-UTR of PCSK9 [10]. In contrast, angiotensin II- (Ang II-) modulated miR-483-3p targets several component proteins in the renin-angiotensin system (RAS), including angiotensin-converting enzyme 1 (ACE-1), AT2R, and ACE-2, implying that this miRNA may be a global modulator of tissue RAS [22]. Of clinical relevance, the serum miR-483 level is decreased in idiopathic PAH patients, acute Kawasaki disease patients, and hyperlipidemic individuals [10, 12, 18].

Because of the beneficial effect of miR-483 in EC function, we speculated that miR-483 exerts a protective role in vascular function under hypertension and that it could be a potential biomarker and therapeutic target for cardiovascular disease. Thus, we tested whether the circulatory level of miR-483 is associated with hypertensive patients. We found that the serum miR-483-3p level was increased in patients with newly diagnosed hypertension, while it decreased in patients with chronic hypertension. The circulating miR-483-3p level is positively correlated with vascular function, and it appears that miR-483-3p can protect against Ang II-activated endothelial and smooth muscle cells via the exosome pathway. We also showed that Ang II receptor blockers induce miR-483, suggesting the therapeutic potential of miR-483.

## 2. Materials and Methods

### 2.1. Patient Selection and Serum Sampling

Our research was approved by the institutional ethics committee of Xi'an No.1 Hospital. All participants provided the written informed consent. The investigation was performed in compliance with the principles of the Declaration of Helsinki for the use of human subjects. Hypertension patients were recruited from Xi'an No.1 Hospital, Xi'an, China, during 2018-2019. The clinical characteristics are presented in Supplemental Tables 1-3. Individuals diagnosed with hypertension met the 2020 Hypertension Clinical Guidelines, i.e., a systolic blood pressure (SBP) of ≥140 mmHg or a diastolic blood pressure (DBP) of ≥90 mmHg [23]. Patients associated with coronary heart disease, diabetes, severe liver or kidney dysfunction, stroke, acute or chronic infectious diseases, and malignant tumors were excluded. Patients, who have taken drugs, such as statins, ACEI, and ARB within the past 6 months, were also excluded.

Patients with hypertension were divided into three cohorts. For cohort 1, 44 patients with newly diagnosed hypertension were recruited, and 21 age-matched healthy controls were enrolled. For cohort 2, 45 patients with a history of hypertension more than 15 years were enrolled, and 40 age-matched controls were enrolled as well. For cohort 3, 40 patients with hypertension receiving telmisartan (80 mg/day) for 3 months were recruited. Whole blood was taken from the cubital vein of patients or controls after overnight fasting. Serum was obtained after centrifugation at 3,000 rpm for 15 min and then aliquoted into separator tubes and stored at -80°C fridge. Flow-mediated dilation (FMD) was measured by the Doppler ultrasound, and the brachial-ankle pulse wave velocity (baPWV) was detected using BP203RPE-IIVP-1000.

### 2.2. Extraction of CD31^+^ Microparticles

CD31^+^ microparticles were extracted as described [11]. Briefly, Dynabeads G (Invitrogen, Carlsbad, CA) were prewashed with PBS. The anti-CD31^+^ antibody (#3528, Cell Signaling Technology) was mixed with prewashed Dynabeads G for 2 hours, following incubation with human serum (1 : 200 dilution) overnight at 4°C. IgG was an isotype control. RNA was extracted using the TRIzol method.

### 2.3. Measurement of Serum NO and ET-1 Levels

Serum levels of NO and ET-1 were measured by ELISA (R&D Systems) as described previously [12]. Serum samples were diluted (1 : 4) using PBS and subsequently incubated in precoated plates for 2 hours at room temperature. After incubation with conjugated secondary antibody for 1 hour, the TMB-ELISA substrate solution was added. Waiting for sufficient color development, 50 *μ*L of stop solution was added to the wells. The plates were recorded at 450 nm on a plate reader (Infinite M200 Pro, Tecan) within 30 min of stopping the reaction.

### 2.4. HUVEC and HASMC Culture

Human umbilical vein endothelial cells (HUVECs) were isolated from the umbilical cord vein using collagenase digestion as described previously [11]. Cells were plated on collagen-coated culture dishes and grown in an M199 medium. HUVECs with passages 5–8 were used for all cell culture studies. Human aortic smooth muscle cells (HASMCs) (ScienCell Co.) were cultured in Dulbecco's modified Eagle medium supplemented with 10% fetal bovine serum and 1% penicillin/streptomycin solution. All cells were cultured under standard cell culture conditions (37°C, 5% CO_2_, and humidified atmosphere).

### 2.5. Transfection of miR-483 Mimic or Pre-miR-483

miR-483 mimic and pre-miR-483 were provided by Shanghai GenePharma Co., China. HUVECs were transfected with 20 nM of miR-483 mimic or pre-miR-483-3p by the use of Lipofectamine 2000 (Invitrogen), following treatment with 200 nM Ang II (MedChemExpress, MCE) for 24 hours.

### 2.6. Isolation, Identification, and Uptake of Exosome

Exosomes were isolated from the supernatants of the in vitro culture medium in HUVECs as described [11]. The supernatants of culture medium were collected when HUVECs had grown to about 90% confluence. After centrifugation at 2,000 g for 10 min and 10,000 g for 30 min, the dead cells and debris were removed. Subsequently, the supernatant was ultracentrifuged at 110,000 g for 70 min at 4°C. The pellet was washed with PBS, centrifuged at 110,000 g for 1 hour, and then filtered using 0.22 *μ*m filters. The isolated exosomes were stored at -80°C fridge until use.

The isolated exosomes were identified through electron microscopy analysis and nanoparticle tracking analysis (NTA) as previously described [24]. For electron microscopy analysis, the exosomes were fixed in 4% paraformaldehyde and then directly placed on a formvar-coated grid. After staining with 2% (*w*/*v*) uranyl acetate, sections were observed and analyzed through transmission electron microscopy (HITACHI, HT7800). To measure the particle size, the exosomes were appropriately diluted in PBS, and the NTA measurement was recorded and analyzed using ZetaView PMX110 (Particle Metrix, Meerbusch, Germany). For exosome uptake, HASMCs were incubated with PKH67- (Sigma-Aldrich) labeled EC-derived exosomes for 24 hours and then fixed in 4% paraformaldehyde for 20 min. Cells were incubated with anti-*α*-SMA antibody (#19245, Cell Signaling Technology) overnight at 4°C, followed by a secondary antibody for 1 hour at room temperature. Images were captured by using a laser scanning confocal microscope.

### 2.7. Real-Time PCR and Western Blotting Analysis

Total RNA and miRNA were extracted from serum samples, CD31^+^ microparticles, HUVECs, and HASMCs by the use of TRIzol reagents (Invitrogen). The relative level of mRNA or miRNA was determined by using SYBR Green or TaqMan probe (Takara). For serum samples, cel-miR-39 (2 nM) was added as a spike-in control. GAPDH and U6 were used as internal controls for normalizing miRNA and mRNA expression. A comparative Ct method, *X* = 2^−ΔΔCt^, was used to determine the relative gene expression [25]. The sequences for real-time PCR primers are presented in Supplemental Table 4. For immunoblotting, proteins were isolated from HUVECs or HASMCs using the RIPA lysis buffer, separated by 4-10% SDS-PAGE, and then transferred to PVDF membranes. After blocking with 5% milk in TBST, the membranes were incubated with the primary antibodies for ACE1 (ab28311, Abcam), TGF-*β* (ab92486, Abcam), CTGF (sc-365970, Santa Cruz), ET-1 (ab2786, Abcam), *β*-actin (sc-47778, Santa Cruz), CD81 (ab79559, Abcam), and CD63 (ab68418, Abcam). The *β*-actin blot was the loading control. The protein bands were observed by enhanced chemiluminescence (Millipore).

### 2.8. Spontaneously Hypertensive Rats

Male normotensive Wistar-Kyoto rats (WKY) and spontaneously hypertensive rats (SHR) (8 weeks old) were purchased from Vital River Laboratory Animal Technology Co., Ltd (Beijing, China). Animals were divided into three groups: WKY controls, SHR plus saline, and SHR plus telmisartan (*n* = 8 rats/group). SHRs were daily intragastrically administered with telmisartan (5 mg/kg) or saline for eight weeks. The SBP of each rat was measured by Visitech Systems BP-2000 (USA). All rats were housed in a pathogen-free facility (12-hour light/dark cycle) and fed a rat chow diet and water ad libitum. Animal experiments were preapproved by the Institutional Animal Ethics Committee of Xi'an Jiaotong University (No. XJTULAC2018.374).

### 2.9. Statistical Analysis

Data analysis was conducted with the GraphPad Prism 8.0 software. Student's *t* test was used for comparison between two groups. One-way ANOVA followed by Tukey's post hoc test was utilized for comparisons between three or more groups. Data were expressed as means ± SEM. *P* values less than 0.05 were considered statistically significant.

## 3. Results

The circulating miR-483-3p level is associated with the progression of hypertension in patients.

**Figure 1:**
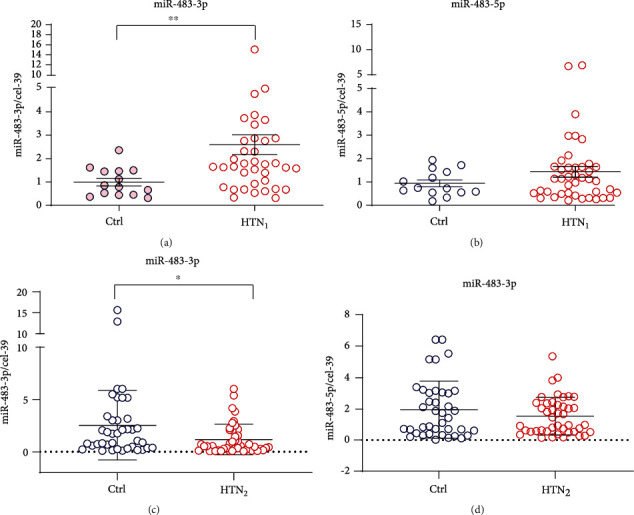
The serum miR-483-3p level is associated with the progression of hypertension in patients. Serum (a) miR-483-3p and (b) miR-483-5p levels in patients with newly diagnosed hypertension (HTN_1_, *n* = 40 − 42) and healthy controls (HC, *n* = 14), measured by qPCR. The qPCR analysis of serum (c) miR-483-3p and (d) miR-483-5p levels in patients with a history of hypertension for more than 15 years (HTN_2_, *n* = 43) and healthy controls (HC, *n* = 39). *Caenorhabditis elegans* miR-39 (cel-39) was used as an internal spike-in control. Data are shown as mean ± SEM. Student's two-tailed *t* test was used for statistical analysis. ^∗∗^*P* < 0.01, ^∗^*P* < 0.05.

To explore whether miR-483 is associated with the development of hypertension, we first compared the levels of miR-483-3p and miR-483-5p in the serum between patients with newly diagnosed hypertension and age-matched healthy controls (cohort 1, *n* = 44 patients). The demographic characteristics of the two groups were shown in Supplemental Table 1. Both SBP (≥140 mmHg) and DBP (≥90 mmHg) of these patients were met with the diagnosis criterion of hypertension. The two groups were comparable in age, sex, smoking history, total cholesterol, and LDL-C, except for body mass index (BMI), triglycerides, and blood glucose (Supplemental Table 1). Serum miR-483-3p levels were substantially increased in hypertension patients in comparison with controls (*P* < 0.01) (Figure 1(a)). However, the miR-483-5p level showed an increased trend, but not with significance (Figure 1(b)). Next, CD31^+^ microparticles were extracted from serum of hypertension patients and healthy controls, respectively. Compared with controls, the miR-483-3p level was much higher in CD31^+^ microparticles from hypertension patients (*P* < 0.01) (Supplemental Fig. 1).

We further validated the serum miR-483-3p and miR-483-5p levels in patients with diagnosed hypertension for more than 15 years (cohort 2, *n* = 45 patients). Other than hypertension, age, sex, and other parameters (total cholesterol, LDL-C, smoking history, etc.) were comparable between the two groups (Supplemental Table 2). Interestingly, we found that serum miR-483-3p levels were significantly lower in patients with long-termed hypertension than that in controls (*P* < 0.05) (Figure 1(c)). However, miR-483-5p levels had no significant change (Figure 1(d)). These data demonstrate that miR-483-3p might be involved in the progression of hypertension.

The serum miR-483-3p level has positive correlation with vascular function.

**Figure 2:**
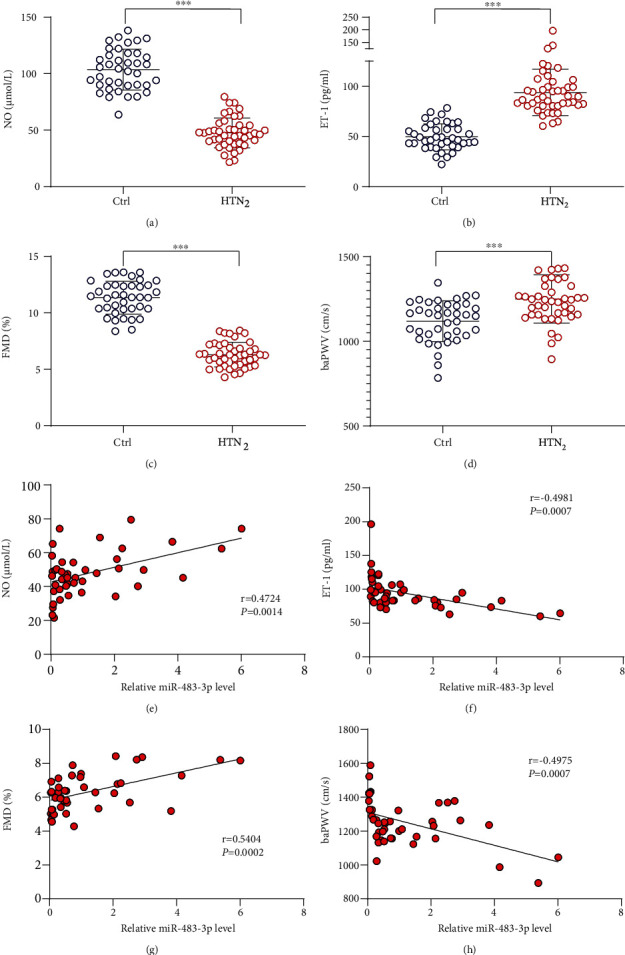
The serum miR-483-3p level has a positive correlation with vascular function. Serum levels of (a) NO and (b) ET-1 (HTN_2_, *n* = 44 − 45; HC, *n* = 39 − 40) measured by ELISA. (c) Flow-mediated dilation (FMD) and (d) brachial-ankle pulse wave velocity (baPWV) were measured for evaluations of endothelial function and vascular damage, respectively. (e–h) The qPCR analysis of miR-483-3p levels in sera collected from cohort 2. The correlation analysis between serum levels of miR-483-3p and those of (e) NO, (f) ET1, (g) FMD, and (h) baPWV was carried out using a nonlinear regression model. Data are shown as mean ± SEM. Significance for data was performed by Student's two-tailed *t* test. ^∗∗∗^*P* < 0.001.

Because of the changed serum miR-483-3p level in hypertension patients, we further investigated whether the serum miR-483-3p level is correlated with the indexes that reflect vascular ECs function, including NO, ET-1, FMD, and baPWV under hypertension. We initially examined these indicators in patients with diagnosed hypertension for more than 15 years (cohort 2, *n* = 45 patients) and found that the serum NO level was remarkably decreased, while the ET-1 level was increased (Figures 2(a) and 2(b)). Consistently, FMD was decreased and baPWV was increased in hypertension patients when compared with controls (Figures 2(c) and 2(d)). The correlation analysis indicated that the serum miR-483-3p level in hypertension patients (cohort 2) was positively correlated with NO and FMD (*r* = 0.4724, *P* = 0.0014 for NO; *r* = 0.5404, *P* = 0.0002 for FMD) (Figures 2(e) and 2(g)), while it negatively correlated with ET-1 and baPWV (*r* = −0.4981, *P* = 0.0007 for ET-1; *r* = −0.4975, *P* = 0.0007 for baPWV) (Figures 2(f) and 2(h)). These results suggest that the serum miR-483-3p level has a positive correlation with vascular function in hypertension individuals.

miR-483-3p protects the endothelium against the Ang II-induced dysfunction.

**Figure 3:**
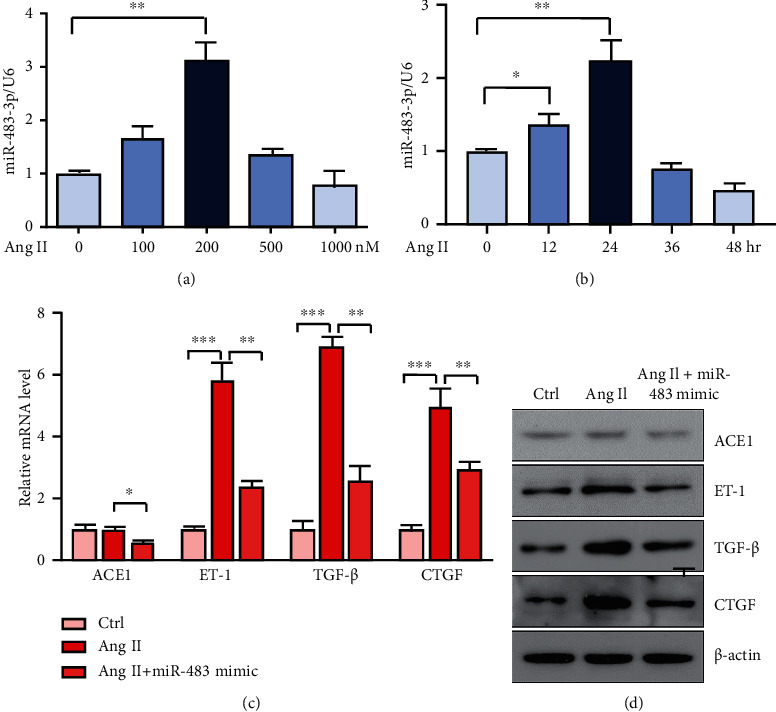
miR-483-3p alleviates Ang II-induced endothelial dysfunction. (a, b) HUVECs were incubated with various concentrations of (a) Ang II (0, 100, 200, 500, and 1,000 nM) for 24 hours or (b) Ang II at 200 nM concentration for different times (0, 12, 24, 36, and 48 hours). miR-483-3p levels in HUVECs were measured by the qPCR analysis. U6 was used as an internal control. (c, d) HUVECs were incubated with Ang II (200 nM) for 24 hours, together with miR-483 mimic (20 nM). (c) The qPCR analysis of mRNA expression of ECs dysfunction-related genes (ACE1, ET-1, TGF-*β*, and CTGF). (d) Western blotting analysis of these ECs dysfunction-related genes. Data are shown as mean ± SEM. Significance for data was carried out by the one-way ANOVA test. ^∗^*P* < 0.05; ^∗∗^*P* < 0.01; ^∗∗∗^*P* < 0.001.

Ang II, a potent vasoconstrictor, induces endothelial dysfunction and hypertension [26]. To discover the function of miR-483-3p in Ang II-induced endothelial dysfunction, HUVECs were treated with Ang II, along with miR-483-3p overexpression. The qPCR analysis showed that the miR-483-3p level was remarkably increased in HUVECs after treatment with Ang II at 200 nM for 24 hours (Figures 3(a) and 3(b)). In ECs, miR-483 targets ET-1, CTGF, and TGF-*β*, which are causal with endothelial dysfunction and fibrogenic response [12, 18]. Ang II strongly induced these ECs dysfunction-related genes, including ACE1, ET-1, TGF-*β*, and CTGF in HUVECs. Expectedly, the addition of miR-483 mimic significantly decreased the mRNA and protein expression of these EC dysfunction genes (Figures 3(c) and 3(d)). This result suggests that the Ang II-induced endothelial dysfunction could be reversed by miR-483, via targeting ACE1, ET-1, TGF-*β*, and CTGF.

Endothelial miR-483 affects smooth muscle cells via the exosome pathway.

**Figure 4:**
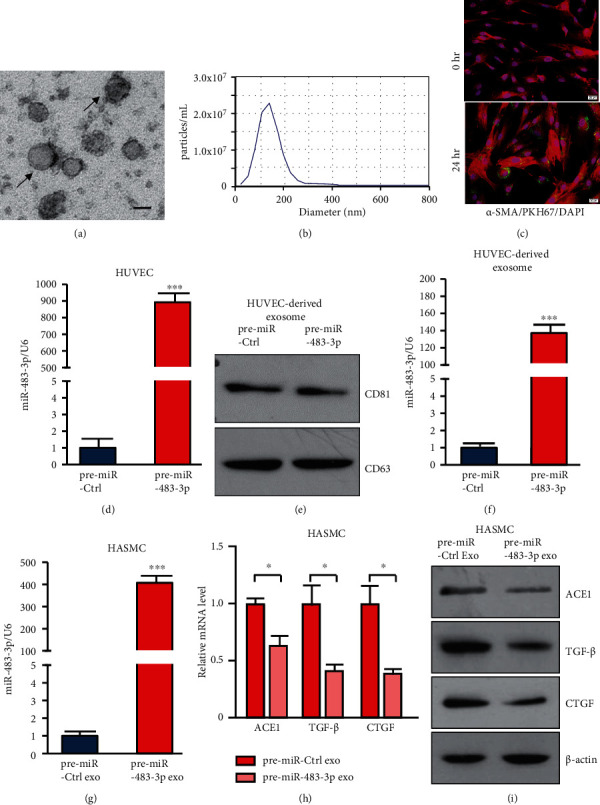
EC-SMC cross talk via exosome-delivered miR-483-3p. (a) A representative electron microscopy image of HUVEC-derived exosomes. Scale bar, 100 nm. (b) A nanoparticle tracking analysis of the size distribution of exosomes. (c) Immunofluorescence images of HASMCs stained with *α*-SMA (red) and DAPI (blue). HASMCs were treated with HUVEC-derived exosomes (PKH67-label, green) for 0 or 24 hours. Laser scanning confocal images are shown. Scale bar, 20 *μ*m. (d) The qPCR analysis of miR-483-3p levels in HUVECs transfected with pre-miR-Ctrl or pre-miR-483-3p for 48 hours. (e) Exosomes isolated from HUVECs were identified by CD81 and CD63 immunostaining. (f) miR-483-3p levels in exosomes isolated from HUVECs transfected with pre-miR-Ctrl or pre-miR-483-3p. (g–i) HASMCs were incubated with HUVEC-derived exosomes for 24 hours. The qPCR analysis of (g) miR-483-3p and ACE1, TGF-*β*, and (h) CTGF in HASMCs. (i) Western blotting analysis of these proteins in HASMCs. Data are shown as mean ± SEM. Student's two-tailed *t* test was used for statistical analysis of data. ^∗^*P* < 0.05; ^∗∗∗^*P* < 0.001.

Carrying various biologically active molecules including miRNAs, exosomes mediate cell-to-cell communication [8]. Because both ECs and SMCs are crucial for vascular remodeling, we hypothesized that miRNA-loaded exosome is an important mechanism to regulate EC-SMC communication. We isolated exosomes from the conditioned media of HUVECs and identified them using an electron microscope and NTA. The exosomes showed a cup-shaped morphology with a size of approximately 100 nm (Figures 4(a) and 4(b)). Furthermore, the PKH67 labeling analysis showed that HUVEC-derived exosomes could be taken by HASMCs (Figure 4(c)). Next, we treated HUVECs with pre-miR-483-3p, which elevated the miR-483-3p levels in HUVECs (Figure 4(d)). The EC-derived exosomes in the medium from HUVECs were further identified by CD81 and CD63 immunoblotting (Figure 4(e)). The content of miR-483-3p was significantly elevated in exosomes from HUVECs with pre-miR-483-3p treatment as compared with pre-miR-Ctrl (Figure 4(f)). These exosomes were then incubated with HASMCs. The levels of miR-483-3p were correspondingly increased in HASMCs, which incubated with exosomes from pre-miR-483-3p-treated HUVECs (Figure 4(g)), while mRNA and protein expression of target genes ACE1, TGF-*β*, and CTGF were remarkably decreased (Figures 4(h) and 4(i)). These results indicate that EC-derived miR-483-3p can be transported to SMCs through the exosome, thus regulating the function of smooth muscle cells.

Antihypertensive drugs induce miR-483-3p in hypertension patients and spontaneously hypertensive rats.

**Figure 5:**
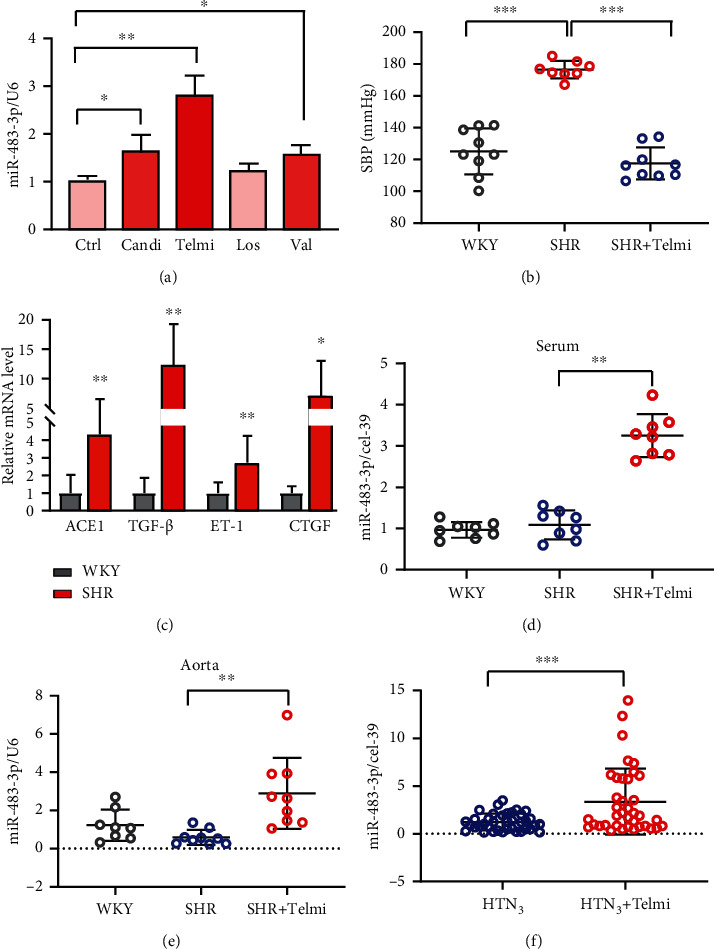
ARBs induce miR-483-3p expression in hypertension subjects and spontaneously hypertensive rats. (a) HUVECs were treated with candesartan (Candi, 2 *μ*M/L), telmisartan (Telmi, 5 *μ*M/L), losartan (Los, 5 *μ*M/L), and valsartan (Val, 5 *μ*M/L) for 24 hours, which was followed by qPCR analysis for miR-483-3p level. U6 was an internal control. (b–e) Spontaneously hypertensive rats (SHR) were administered intragastrically with saline or telmisartan (5 mg/kg/day) for 8 weeks. Homologous Wistar-Kyoto (WKY) rats were considered the control group. (b) Systolic blood pressure (SBP). (c) Relative mRNA expression of ACE1, ET-1, TGF-*β*, and CTGF in the arteries of SHR. (d) Serum and (e) aortic miR-483-3p levels in SHR treatment with telmisartan. (f) Serum miR-483-3p levels in hypertension patients who orally took telmisartan (80 mg/day) before or after 3 months. Data are indicated as mean ± SEM. The one-way ANOVA test was used for statistical analysis. ^∗^*P* < 0.05; ^∗∗^*P* < 0.01; ^∗∗∗^*P* < 0.001.

Angiotensin II receptor blockers (ARBs) are the most commonly used blood pressure-lowering drugs, including candesartan, telmisartan, losartan, and valsartan [27]. Given that miR-483-3p has a protective role in hypertension, we speculate that it may be involved in the molecular mechanism of ARBs to improve vascular function under hypertension. The qPCR analysis indicated that the miR-483-3p level was remarkably increased in HUVECs incubated with candesartan, telmisartan, and valsartan, but not losartan (Figure 5(a)). Because telmisartan exhibited the strongest effect on the induction of miR-483-3p, we selected telmisartan as a representative ARBs for further studies. SHRs were intragastrically administered telmisartan (5 mg/kg) or saline for eight weeks. Compared with WKY, the SBP of SHR was substantially elevated, following upregulation of ACE1, TGF-*β*, ET-1, and CTGF genes (Figures 5(b) and 5(c)). After treatment with telmisartan, SBP was significantly decreased as compared with the saline group (Figure 5(b)). Correspondingly, miR-483-3p levels both in the serum and aorta were markedly increased in the telmisartan group (Figures 5(d) and 5(e)). Furthermore, we compared the miR-483-3p level in hypertension patients before (cohort 3, *n* = 40 patients) and after (cohort 3, *n* = 38 patients) telmisartan treatment (80 mg/day) for three months. Both SBP and DBP were significantly decreased after telmisartan treatment, while other variables were comparable in cohort 3 (Supplemental Table 3). The serum miR-483-3p level was dramatically elevated in hypertension patients after telmisartan treatment (Figure 5(f)). These results suggest ARBs, at least telmisartan, increased the level of miR-483-3p in hypertension patients.

## 4. Discussion

**Figure 6:**
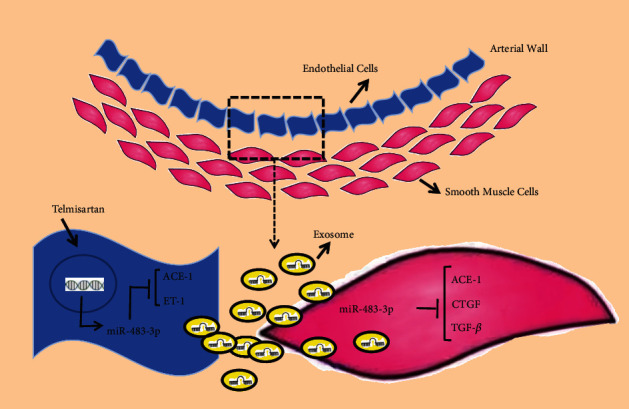
Summary of the study. Antihypertensive drug telmisartan-induced miR-483-3p in ECs improves endothelial function. Also, EC-derived miR-483-3p taken up by SMCs can potentiate vascular function to decrease the hypertension-induced vascular damage.

The main finding of the present study is the association between the circulating miR-483-3p level and the progression of hypertension, and its positive correlation with vascular function. Serum levels of miR-483-3p are upregulated in patients with first-diagnosed hypertension, while they are downregulated in patients with long-term hypertension. Furthermore, we found that miR-483-3p protected against Ang II-induced endothelial dysfunction via suppressing the expression of genes related to endothelial dysfunction. Endothelial miR-483-3p, when released into exosomes, can be taken up by neighboring SMCs in the artery, contributing to regulate phenotypic target genes (ACE-1, CTGF, and TGF-*β*) in these cells (summarized in Figure 6). In vivo, miR-483-3p expression can be induced in patients with hypertension and SHR animal model, who are treated with the antihypertensive drugs. Collectively, our findings suggest that miR-483-3p has protective effects on vascular function during the onset of hypertension. It may be considered a potential therapeutic target for hypertension-related cardiovascular diseases.

Hypertension is closely related to obesity and metabolic syndromes, impacting 26.4% of the global population [28, 29]. Increasing data have shown that abnormal miRNAs expression is related to pathogenesis or organ damages of hypertension [30]. In our study, to analyze the relationship between miR-483 levels and the development of hypertension, whole blood and characteristic data were obtained from patients with newly diagnosed hypertension as well as patients with a history of hypertension for more than 15 years. In cohort 1, we found that the serum miR-483-3p level, but not miR-483-5p, was significantly increased in newly diagnosed hypertension individuals as compared with those not (*P* < 0.01), suggesting miR-483-3p has a protective role during the early stage of hypertension. Gallo et al. reported that miR-483-5p is a major therapeutic target for cardiometabolic disease because it correlates with waist circumference, high-density lipoprotein, and BMI [31]. Our study showed that although BMI, triglyceride, and blood glucose of hypertension individuals were higher than that of healthy individuals, the levels are still under the borderline of obesity, hyperglycemia, and dyslipidemia. However, these variables, including obesity, hyperglycemia, and dyslipidemia might affect the circulating miR-483-3p or miR-483-5p levels. Regarding the miR-483 origin in the serum of hypertension patients, the data from CD31^+^ microparticles of human serum suggest that miR-483-3p was secreted from vascular ECs at least partly (Supplemental Fig. 1). Therefore, the change of miR-483-3p level in hypertension patients is closely associated with endothelial dysfunction.

In cohort 2, we found an indeed decreased serum level of miR-483-3p in long-term hypertension patients along with vascular EC and SMC dysfunction, which was supported by the increased level of ET-1 and baPWV, and decreased level of NO and FMD. With respect to endothelial biology, it has been shown that miR-483-3p protects against endothelial-mesenchymal transition and inflammation in human aortic valve endothelial cells in a flow-dependent manner [32]. Recent study reported that renin-angiotensin-aldosterone system-related miR-483-3p is reduced in patients with essential hypertension [33]. With respect to the opposite change of miR-483-3p between cohort 1 and cohort 2, we give an explanation as follows. At the beginning of hypertension, the expression of IGF-2 is induced under pathological state [34], increasing the miR-483-3p level in endothelial cells. EC-derived miR-483-3p secretes into exosomes and then is taken up by the neighboring smooth muscle cells, inhibiting its target genes and protecting from the aortic stiffness. Subsequently, the upregulation of miR-483-3p exerts a feedback inhibition on the IGF-2/miR-483 expression, thereby decreasing miR-483. Alternatively, a compensating mechanism induced by early onset of hypertension may elevate the miR-483 level in ECs. During the development of hypertension with the aggravated ECs and SMCs dysfunction, the level of miR-483-3p further declines, unable to maintain vessel homeostasis.

Of note, we revealed a positive correlation between miR-483 and EC and SMC function in data from cohort 2, which was supported by a forward correlation between miR-483 and NO, FMD, and a reverse correlation between miR-483 and ET-1, baPWV. These results are consistent with previous reports that serum miR-483 levels in idiopathic PAH patients are decreased and that increased miR-483 levels are protective [12, 18]. The protective role of miR483 could be further supported by the induced miR-483-3p level in patients with hypertension and SHR animal model after treatment with antihypertensive ARBs. Because EC dysfunction and arterial remodeling are key pathophysiological features for hypertension, miR-483 may be considered a novel diagnostic target for the progression of hypertension.

EC dysfunction is associated with the increased proinflammatory cytokines, more production of vasoconstrictors, and less production of vasodilators [35]. Previous study has shown that miR-483 can improve EC function through alleviating inflammation and fibrogenesis and thus protect against vascular injury and aneurysm formation in acute vasculitis [18]. In the current study, we incubated HUVECs with Ang II in vitro to mimic pathophysiologic conditions of hypertension and endothelial dysfunction. Using this approach, we found that miR-483 overexpression could reverse the Ang II-elevated ACE1, ET-1, CTGF, and TGF-*β* (Figures 3(c) and 3(d)). These findings further support that exogenously administered miR-483 may help attenuate the vascular impairments in hypertension.

The information exchange between ECs and SMCs is crucial for vascular structure and function [8, 36]. Exosomes mediate cell-to-cell communications via derived biologically active molecules including microRNAs and therefore are likely to participate in endothelial dysfunction in cardiovascular disease [37, 38]. To investigate how miR-483 transport between ECs and SMCs, we extracted exosomes in the conditioned medium collected from HUVECs with miR-483 overexpression, which was then used to treat HASMCs. The consequent increase of miR-483-3p in HASMCs along with downregulation of vascular dysfunction-related genes suggest that EC-derived miR-483 could transfer to SMCs and contribute to SMC phenotypic presentations. Thus, therapeutic delivery of miR-483 mimics via engineered exosomes may be an optimal strategy for therapeutic use of miR-483-3p to treat hypertension-related cardiovascular diseases.

## Data Availability

The datasets generated during and/or analyzed during the current study are available from the corresponding author on request.

## Supplementary Materials

Supplementary Materials Figure S1: the miR-483-3p level is elevated in CD31^+^ microparticles from the serum of hypertension patients. Table S1: clinical characteristics of healthy control and patients with newly diagnosed hypertension. Table S2: clinical characteristics of healthy control and patients with diagnosed hypertension for more than 15 years. Table S3: clinical characteristics of hypertension patients before and after telmisartan treatment. Table S4: primers used for miRNA and mRNA qPCR.
