# Supplementary material for: Endothelial MicroRNA-483-3p Is Hypertension-Protective
Source: Oxid Med Cell Longev. 2022 Feb 17;2022:3698219. doi: 10.1155/2022/3698219 (PMC8872655; doi:10.1155/2022/3698219)
Supplement: Supplementary Materials — Figure S1: the miR-483-3p level is elevated in CD31+ microparticles from the serum of hypertension patients. Table S1: clinical characteristics of healthy control and patients with newly diagnosed hypertension. Table S2: clinical characteristics of healthy control and patients with diagnosed hypertension for more than 15 years. Table S3: clinical characteristics of hypertension patients before and after telmisartan treatment. Table S4: primers used for miRNA and mRNA qPCR. [file 3698219.f1.doc]

**
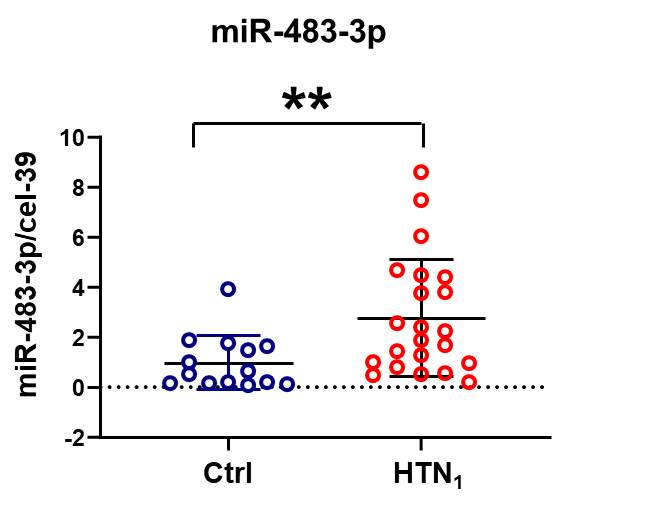
**

**Supplemental Fig.1 miR-483-3p level is elevated in CD31+ microparticles from serum of hypertension patients.** CD31+ microparticles were isolated from serum of hypertension with newly diagnosed patients (n = 22) and controls (n = 14). The levels of miR-483-3p were measured by qPCR.

**Supplemental Table 1. Clinical characteristics of healthy control and patients with newly diagnosed hypertension**

|  | Control  n=21 | Hypertension  n=44 | *P* value |
| --- | --- | --- | --- |
| Age (years) | 41.05±1.81 | 45.34±1.48 | 0.09 |
| Sex (Male) | 8 (38%) | 27 (61.4%) | 0.0784 |
| Current smoker | 6 (28.57%) | 13 (29.55%) | 0.936 |
| SBP (mmHg) | 124.4±2.44 | 151.6±1.41 | <0.0001 |
| DBP (mmHg) | 75.38±1.59 | 99.09±2.69 | <0.0001 |
| BMI (kg/m^2^) | 22.76±0.84 | 26.04±0.66 | 0.0045 |
| Total Cholesterol (mmol/L) | 4.84±0.17 | 5.02±0.13 | 0.41 |
| Triglycerides (mmol/L) | 1.22±0.16 | 2.02±0.17 | 0.001 |
| LDL-C (mmol/L) | 2.33±0.12 | 2.48±0.07 | 0.26 |
| Blood Glucose (mmol/L) | 5.07±0.13 | 5.98±0.21 | 0.0002 |

**Supplemental Table 2. Clinical characteristics of healthy control and patients with diagnosed hypertension for more than 15 years**

|  | Control  n=40 | Hypertension  n=45 | *P* value |
| --- | --- | --- | --- |
| Age (years) | 59.43±10.33 | 61.64±7.95 | 0.2673 |
| Sex (Male) | 12 (30%) | 16(35.5%) | 0.5865 |
| Current smoker | 8 (20.00%) | 13 (29.55%) | 0.3738 |
| SBP (mmHg) | 127.4±7.49 | 159.0±15.83 | <0.0001 |
| DBP (mmHg) | 76.38±5.47 | 82.32±6.82 | <0.0001 |
| BMI (kg/m^2^) | 24.62±5.84 | 25.63±6.92 | <0.0001 |
| Total Cholesterol (mmol/L) | 4.23±0.82 | 4.27±0.13 | 0.8745 |
| Triglycerides (mmol/L) | 1.41±0.74 | 1.38±0.52 | 0.8160 |
| LDL-C (mmol/L) | 2.70±0.58 | 2.71±1.05 | 0.9677 |
| Blood Glucose (mmol/L) | 4.99±1.03 | 5.12±0.95 | 0.5763 |

**Supplemental Table 3. Clinical characteristics of hypertension patients before and after telmisartan treatment**

|  | Before treatment  n=40 | After treatment  n=38 | *P* value |
| --- | --- | --- | --- |
| Age (years) | 61.40±10.00 | 62.35±9.69 | 0.7075 |
| Sex (Male) | 20 (8) | 18 (8) | >0.9999 |
| Current smoker | 1 (5.00%) | 1 (5.56%) | >0.9999 |
| SBP (mmHg) | 165.3±6.18 | 127.89±5.39 | <0.0001 |
| DBP (mmHg) | 92.59±9.64 | 71.33±15.a | <0.0001 |
| Total Cholesterol (mmol/L) | 3.83±0.76 | 4.06±0.59 | 0.9106 |
| Triglycerides (mmol/L) | 1.52±0.66 | 1.31±0.58 | 0.0751 |
| LDL-C (mmol/L) | 2.33±0.71 | 2.69±0.49 | 0.8428 |
| Blood Glucose (mmol/L) | 5.01±0.79 | 4.89±0.59 | 0.3246 |

**Supplemental Table 4. Primers used for miRNA and mRNA qPCR**

| Name | Species | Sequence |
| --- | --- | --- |
| ACE1 | *Homo sapiens* | Forward: GGAGGAATATGACCGGACATCC  Reverse: TGGTTGGCTATTTGCATGTTCTT |
| ET-1 | *Homo sapiens* | Forward: GGGCTGAAGACATTATGGAG  Reverse: CGAAGGTCTGTCACCAATGT |
| TGF-β | *Homo sapiens* | Forward: TACCATGCCAACTTCTGCCT  Reverse: ACGATCATGTTGGACAGCTG |
| CTGF | *Homo sapiens* | Forward: TTACCAATGACAACGCCTCC  Reverse: GTACGGATGCACTTTTTGCC |
| GADPH | *Homo sapiens* | Forward: ACCACAGTCCATGCCATCAC  Reverse: TCCACCACCCTGTTGCTGTA |
| | miR-483-3p | | --- | | *Homo sapiens* | | Forward: ACACTCCAGCTGGGATCACTCCTCCCCT  Reverse: CTGGTGTCGTGGAGTCGG | | --- | |
| | miR-483-5p | | --- | | Homo sapiens | | | Forward: ACACTCCAGCTGGGAAAGACGGGAGGAAA  Reverse: CTGGTGTCGTGGAGTCGG | | --- | | | --- | --- | |
| | miR-483-3p | | --- | | | *Rattus norvegicus* | | --- | | | Forward: CACTCCAGCTGGGACACTCCTCCCCTCCCG  Reverse: CTGGTGTCGTGGAGTCGG | | --- | |
| Cel-39 |  | Forward: ACACTCCAGCTGGGAUCACCGGGUGUAAA  Reverse: CTGGTGTCGTGGAGTCGG |
| U6 |  | Forward: ACACTCCAGCTGGGAUCACCGGGUGUAAA  Reverse: AACGCTTCACGAATTTGCGT |
